# Immune Response to the Recombinant Apa Protein from *Mycobacterium tuberculosis* Expressed in *Streptomyces lividans* After Intranasal Administration in Mice. Induction of Protective Response to Tubercle Bacillus Aerosols Exposure

**DOI:** 10.1007/s00284-024-03697-7

**Published:** 2024-05-30

**Authors:** José Alberto Martínez-Sotelo, Antonio J. Vallecillo, Cristina Parada, Erika Segura, Jaime Campuzano, Mayra Silva-Miranda, Luis Servín-González, Clara Espitia

**Affiliations:** 1 Departamento de Inmunología, Instituto de Investigaciones Biomédicas, Universidad Nacional Autónoma de México, C.P. 04510 Mexico City, Mexico; 2 Escuela de Medicina Veterinaria y Zootecnia, Facultad de Ciencias Agropecuarias, Universidad de Cuenca, C.P. 010205 Cuenca, Azu Ecuador; 3 Departamento de Patología, Facultad de Medicina Veterinaria y Zootecnia, Universidad Nacional Autónoma de México, C.P. 04510 Mexico City, Mexico; 4 Catedrática CONAHCYT, Departamento de Inmunología, Instituto de Investigaciones Biomédicas, Universidad Nacional Autónoma de México, C.P. 04510 Mexico City, Mexico; 5 Departamento de Biología Molecular y Biotecnología, Instituto de Investigaciones Biomédicas, Universidad Nacional Autónoma de México, C.P. 04510 Mexico City, Mexico

## Abstract

Identifying and evaluating potential vaccine candidates has become one of the main objectives to combat tuberculosis. Among them, mannosylated Apa antigen from *Mycobacterium tuberculosis* and the non-mannosylated protein expressed in *Escherichia coli*, have been studied. Although both proteins can induce a protective response in mice, it has been considered that native protein can be dispensed. In this work, we study the protective response induced by Apa expressed in *E. coli* and in *Streptomyces lividans.* The latter*,* like native is secreted as a double band of 45/47 kDa, however, only its 47 kDa band is mannosylated. Both antigens and BCG were intranasal administrated in mice, and animals were then challenged by aerosol with *M. tuberculosis* H37Rv. The results showed that both, Apa from *S. lividans* and *E. coli* conferred statistically significantly protection to animals compared to controls. The cytokine immune response was studied by an immunoassay after animals’ immunization, revealing that Apa from *S. lividans* induced a statistically significant proliferation of T cell, as well as the expression of IFN-γ, IL-1β, IL-17 and IL-10. In contrast, non-proliferation was obtained with non-mannosylated protein, but induction of IL-12 and IL-17 was observed. Together, these results demonstrate that both proteins were able to modulate a specific immune response against *M. tuberculosis,* that could be driven by different mechanisms possibly associated with the presence or not of mannosylation. Furthermore, stimulation of cells from BCG-vaccinated animals with the proteins could be an important tool, to help define the use of a given subunit-vaccine after BCG vaccination.

**Supplementary Information:**

The online version contains supplementary material available at 10.1007/s00284-024-03697-7.

## Introduction

Tuberculosis is a disease caused by *M. tuberculosis* and is one of the leading causes of morbidity and mortality in the world with 10.4 million new cases and 1.4 million deaths in 2021. The disease was considered a global public health emergency since 1993 by the World Health Organization [1]. Currently, the situation has worsened due to the emergence of multi and extensively drug resistant strains, Human Immunodeficiency Virus (HIV)-Tuberculosis co-infection and type 2 diabetes epidemic, moreover, the disease is difficult to diagnose, needs a long treatment and the access to medical services is insufficient [1–3].

At present, there is only one tuberculosis vaccine licensed for human use, the Bacillus Calmette-Guérin (BCG), which administered in children under 5 years of age provides strong protection against the severe forms of the disease (meningeal and milliary) for up to 10 years, however, its efficacy declines with each consecutive year. Moreover, the efficacy of BCG varies from 0 to 80% in adult pulmonary tuberculosis. The BCG vaccine was first produced in 1921, and because of its limitations a better vaccine is necessary [3, 4]. Several vaccines against *M. tuberculosis* are being proposed, some of which are already being evaluated in the preclinical and clinical phases [5]. Different strategies have been used in the design of new vaccines against tuberculosis, such as BCG transformed with genes that express immunodominant antigens, the restoration of BCG genes lost during the attenuation of the strain [6] and transfection of BCG with genes essential for protection such as IL-2, IFN-γ or IL-18 [7]. Other approaches are the production of *M. tuberculosis* live attenuated vaccines by mutation of genes coding for virulence factors or the use of non-living vaccines such as the *M. tuberculosis* immunodominant antigens (subunit-vaccines) and DNA vaccines [3, 8, 9].

Among the protein antigens that have the potential to be subunit-vaccines are the proteins with post-translational modifications. The presence of methylation for instance, in the hemagglutinin binding heparin adhesin (HbhA) of *M. tuberculosis* was important for the induction of a strong IFN-γ response that confers protection against *M. tuberculosis* infection, compared with the lack of protection of the animals immunized with the recombinant protein expressed in *E.coli* [10], or the presence of glycosylation in the Alanine-proline rich-protein (Apa, Rv1860), a 47/45 kDa secreted molecule, one of the first glycoproteins described in *M. tuberculosis* [11], since it was found that mannosylation of this protein was necessary for T cell proliferation in mice [12, 13].

Furthermore, it had been demonstrated that immunization of guinea pigs with Apa DNA vaccine and boosted with recombinant Apa expressed in a poxvirus conferred protection against *M. tuberculosis* challenge [14]. In a comparative study, both native glycosylated Apa and non-glycosylated expressed in *E. coli*, were used as recombinant subunit-vaccines in BCG-vaccinated mice. The results showed that although both forms conferred protection against a *M. tuberculosis* challenge, non-mannosylated protein was preferentially recognized over native mannosylated. Important to mention, it was also determined that Apa imparts significant protection in elderly mice and improves waning BCG immunity [15, 16].

In the present work, we studied the immune response to recombinant S*. lividans* Apa secreted into the culture medium as a complex formed by two bands, one of them modified with mannoses in the same amino acid positions as the native protein [17–19] and a second band non-glycosylated. It should be noted that, recombinant *M. tuberculosis* proteins with post-translational modifications such as glycosylation or methylation, outside of Mycobacterium genus, have been only produced in bacteria phylogenetically related to *M. tuberculosis* such as *S. lividans* and *Rhodococcus* e*rythropolis*, with the advantage that recombinant proteins expressed in these bacteria can be obtained on a large scale and under controlled conditions [19–22]. Since members of the genus Streptomyces do not synthesize lipoglycan structures like Mycobacterium it constitutes an advantage for the purification of glycosylated mycobacterial proteins produced in this bacterium [23]. Both, mannosylated and non-mannosylated antigens were intranasally (i.n.) administered to mice. This route of administration is a non-invasive procedure that could offer a better protection against *M. tuberculosis* infection compare with subcutaneous or oral route. In addition, this route induces both a systemic and mucosal immune response playing an important role in the kind of immune response elicited [24–26]. Aerosol route was used for mice infection with *M. tuberculosis,* such route allows a more effective evaluation of the protector effect of vaccine candidates, since it has been described that modification of the pathogen invasion route could modify the mechanisms that are activated to combat them [27–29]. The aerosol mice infection with *M. tuberculosis* using the Glas-Col chamber has been widely studied and standardized. It has demonstrated that in mice exposed to a predetermined dose of a *M. tuberculosis* strain for 60 min in the inhalation aerosol chamber, initial infectious doses of 450–500 CFU of H37Ra or 150–200 CFU of H37Rv strains were achieved [30]. Also, in animals aerosolized with *M. tuberculosis* H37Rv after 6 weeks post-vaccination challenge, approximately 200 bacteria were deposited in the lungs of each animal [31].

Under those immunization conditions the cytokine immune response induced for either *E. coli* non-glycosylated Apa and the *S. lividans* mannosylated/non-mannosylated mix protein was determined as well as their capacity to protect the animals against *M. tuberculosis* infection.

## Materials and Methods

### Mice

Sixty pathogen-free, 6–8 weeks old female BALB/c mice purchased from the animal house of Instituto de Investigaciones Biomédicas, (IIBo) UNAM (México), were used in this study. Two vaccination experiments with 30 animals each were carried out in a Biosafety Level II and III animal facilities, as required for the experiments. Animals were fed on standard diet and water. All procedures were approved by the Institutional COMMITTEE FOR THE CARE AND USE OF LABORATORY ANIMALS (Protocol ID 180).

### Antigens

#### Recombinant *S. lividans* Apa Protein

Recombinant Apa expressed in *S. lividans* 66 strain 1326 (r*S.lividans*Apa) was recovered from the culture supernatant as described before with some modifications [19]. To obtain the r*S.lividans*Apa, bacterial culture supernatant was precipitated with Ammonium sulfate (J.T. Baker, USA) 45% saturation at 4 °C overnight (ON) and centrifuged at 9700×*g* for 30 min at 4 °C. The precipitate obtained was resuspended in minimum volume of Phosphate buffered saline (PBS) pH 7.4, and dialyzed against Sodium acetate buffer 0.1 M, pH 5 ON at 4 °C, after centrifugation at 9700×*g* for 15 min the precipitate was resuspended with Tris-HCl (J.T. Baker) 0.02 M pH 8.3 and dialyzed with the same buffer ON at 4 °C. Sample was centrifugated at 9700×*g* for 15 min at 4 °C and passed through a Sepharose HiTrap-Q, (GE Healthcare Biosciences, Pittsburgh, PA, USA) anion exchange chromatography column as described below.

### Cloning, Expression of *M. tuberculosis* Apa in *E. coli*, Purification as N-Terminal His-Tagged Protein

The coding region of the *Apa* gene was amplified by PCR with high fidelity DNA polymerase *Pfx* (Invitrogen, Carlsbad, USA) from *M. tuberculosis* H37Rv genomic DNA with the oligonucleotide primers *Apa*15bF (5′-GCATATGGATCCGGAGCCAGCGCC-3′) *Apa*15bR (5′GCTGATCAGGCCGGTAAGGTCCGC-3′) (NdeI site and BclI site underlined). The PCR product (873 bp) was cloned into the pCR4 Blunt-TOPO vector (Invitrogen) with the use of TOP10F’ strain. Vector was digested with NdeI and BclI and the released fragment was gel-purified and ligated into the NdeI and BamHI (BclI generated end is compatible with BamHI end) of pET15b (Novagen Inc, Madison, WI, USA). The identities and orientation of the inserts were confirmed by restriction analysis and DNA sequencing. *E. coli* Rosetta (DE3) (Novagen) was heat shock-transformed with pET15b-Apa. A single recombinant colony was grown in LB (Difco, Sparks, MD, USA)/supplemented with 100 µg mL^−1^ of Carbenicillin (Invitrogen) (LB/Car ON at 37 °C with shaking (200 r.p.m). Next day, ON culture was diluted 1/100 in LB/Car and incubated at 37 °C with shaking until OD_600nm_ reached ~ 0.4. Then, the culture was induced with 250 µM (final concentration) of Isopropyl-β-D-1-galactopyranoside (IPTG) (Roche, Applied Science, Mannheim, Germany) and kept for 4 h at 37 °C. Cells were collected by centrifugation, resuspended in 50 mM Tris-HCl, 50 mM NaCl, 20 mM Imidazole, pH 8.0, to obtain the soluble extract (SE), and histidine-tagged r*E.coli*Apa was purified from this fraction in an AKTA FPLC (GE Healthcare Biosciences). using a HisTrap HP de 1 ml (GE Healthcare previously equilibrated with 50 mMTris-HCl, 50 mM NaCl, 20 mM imidazol, pH 8.0. Protein was eluted with 50 mM Tris-HCl, 50 mM NaCl, 500 mM imidazol, pH 8.0 at 1 ml/min, using a gradient 10, 30, 50 y 100% of imidazol. Finally, to eliminate the *E. coli* LPS, protein was passed through a Sepharose HiTrap-Q anion exchange chromatography column [32] which was previously washed with dH_2_O and 10 volumes of Tris-HCl 20 mM pH 8.3 and eluted with NaCl gradient. Proteins were dialyzed against PBS pH 7.4 and quantified by BCA kit (Thermo Fisher, Waltham, MA, USA). The amount of endotoxin in the recombinant protein was determined using Pierce, LAL Chromogenic Endotoxin Quant Kit (Thermo Scientific, Ilinois USA), according to the manufacturer's, instructions. Endotoxin analysis of the protein was 0.133 EU/ml. Recombinant proteins were stored at − 70 °C until use.

### Mycobacterial Strains

*M. tuberculosis* H37Rv ATCC 27294 and BCG Phipps from Instituto de Investigaciones Biomédicas collection were grown in Middlebrook 7H9 liquid medium (Difco, sparks, MD, USA) supplemented with 10% ADC (Difco, USA) and Tiloxapol 0.02% (Sigma-Aldrich, St. Louis, Mo, USA). Bacteria aliquots with OD_600nm_ = 0.4 were frozen at -70 °C. For aerosol infection, one aliquot was grown up to obtain OD_600nm_ = 0.9 and 10 mL were used in a whole-body inhalation exposure system (Glas-Col, LLC). CFU were determinate by plating serial dilutions in 7H11 agar plates supplement with 10% OADC (Difco, Sparks, MD, USA), (4.8 × 10^6^ CFU/mL).

For BCG Phipps, aliquots of OD_600nm_ = 0.9 were frozen at – 70 °C and CFU were determinate by plating serial dilutions in 7H11 agar plates supplement with 10% OADC (Difco), (1.4 × 10^6^ CFU/mL).

### Vaccination of Mice

Six mice were i.n vaccinated, with 20 μL of BCG Phipps (7 × 10^5^ CFU) to external nares (10 μL by nostril) using a micropipette fine-tip and allowing the mouse to inhale the suspension into the lungs naturally. For recombinant Apa proteins vaccination, six mice received by i.n route 3 doses at 2 weeks intervals of 10 μg of recombinant Apa proteins per dose, proteins were emulsified in a DDA-MPL adjuvant (Sigma-Aldrich) (250 μg DDA and 25 μg MPL/dose). Both methods were carried out as described previously [33]. The control groups (six mice per group) were administrated i.n. with 20 μL of PBS or 20 μL of DDA-MPL adjuvant (250 μg DDA and 25 μg MPL/dose). These adjuvants were used, since it had been reported that DDA promotes protective immunity in mice when tested as an adjuvant for a tuberculosis subunits vaccine [34], and significant improvement was achieved when the inflammation-promoting molecule monophosphoryl lipid A (MPL) was added to a DDA [35]. This experiment was duplicated.

### *Mycobacterium tuberculosis* Mice Infection

After four weeks of the last dose of recombinant Apa proteins or 8 weeks of BCG vaccination, three mice of each group were challenge by aerosol route using 10 mL of a suspension of 4.8 × 10^6^ CFU/mL of *M. tuberculosis* H37Rv strain in a whole-body inhalation exposure system (Glas-Col, LLC, USA), the infection was carried out as describe previously [36].

The CFU levels were evaluated at 6 weeks post infection. The bacillary burden was determined by plating lung homogenates onto the Middlebrook 7H11/OADC (Difco). The CFU were enumerated after 4 weeks of incubations at 37 °C and numbers were expressed as the log_10_ values of the geometry mean for three mice.

### Lymphocyte Proliferation

After four weeks of the last dose of recombinant Apa proteins or 8 week of BCG vaccination, three animals were sacrificed, and the spleens were aseptically removed. Cells isolated from spleen were stained with carboxyfluorescein succinimidyl ester (CFSE) 0.5 μM as a final concentration. Stained lymphocytes were seeded in sterile 96-well flat-bottom tissue culture plates (Costar, USA) at 1 × 10^5^ cells per well in 150 μL of supplemented RPMI-1640. Stained lymphocytes were seeded in sterile 96-well flat-bottom tissue culture plates (Costar) at 1 × 10^5^ cells per well in 150 μL of supplemented RPMI-1640 (Gibco, Grand Island, NB, USA). For each treatment group, cells were stimulated in triplicate with 10 μg of recombinant Apa proteins, Concanavalin A (2 µg/mL) or BCG Phipps strain at multiplicity of infection MOI of 10:1 each one in 50 μL of supplemented RPMI-1640. Con-A (Invitrogen) as a positive control for cell viability and reactivity evaluation and medium alone as a negative control were employed. All cells were incubated at 37 °C for 120 h. in a humid atmosphere containing 5% CO_2_.

Once the incubation was completed, the lymphocytes were harvested and their surface was stained with CD3-PE-Cy7, CD4-PE and CD8-APC antibodies (Invitrogen). The stained cells were acquired on the Blue/Red ATTUNE Cytometer and analyzed using FlowJo software (Treestar, Inc.). Lymphocytes were identified based on their scatter patterns so CD4 T cell were considered as PE-Cy7+/PE+/APC-. The proliferative cells were identified between the cells cultured alone (CFSE intensity of non-divided cells) and non-label cells (auto-florescence) and were expressed as percentage of proliferation. Prior to cytometric analysis, the cells stimulated with BCG were fixed with 1% paraformaldehyde (Sigma Aldrich).

### Multiplex Microsphere-Based Cytokine Immunoassay

The supernatants of lymphocyte cultures stimulated with the different treatments were harvested by centrifugation after 120 h of incubation and stored at – 70 °C. Supernatants were subsequently analyzed in duplicated using a Bio-Plex Cytokine Assay (R&D Systems, INC, USA) according to the manufacturer’s instructions. The Median fluorescence intensity (MFI) was determinate on Bio-Plex 100 instrument. The concentrations for IL-2, IFN-γ, TNF-α, IL-12p70, IL-1β, IL-17 and IL-10 are reported as pg/mL.

### Statistical Analysis

The data were analyzed using analysis of variance (ANOVA). Differences between groups in CFU’s levels, proliferation percentage or cytokines concentrations were assessed by a one-way ANOVA with Tukey’s correction (GraphPad Prism version 9). *P* values < 0.05 were considered statistically significant.

## Results

### Expression and Purification of Recombinant Apa Proteins from *S. lividans *and *E. coli*

**Fig. 1:**
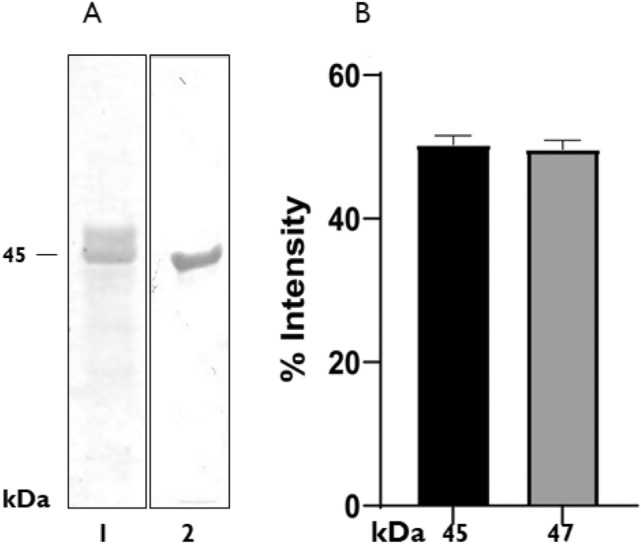
Expression of recombinant Apa proteins from *Streptomyces lividans* and *Escherichia coli.* **a** Coomassie blue staining of purified proteins (lane 1 and 2, respectively). **b** Each protein band (45 and 47 kDa) was analyzed using *ImageJ* software. The results represent 3 independent experiments

Recombinant Apa expressed in *S. lividans* 66 strain 1326 was obtained from culture supernatant and purified as described above. *E. coli* Rosetta (DE3) heat shock-transformed with pET15b*-Apa* expressed the His-tagged recombinant protein, which was purified from soluble bacterial extract by affinity and anion exchange chromatography (Suppl. Figure 1). SDS-PAGE resolved recombinants proteins were transferred to PVDF membranes and stained with Coomassie blue for visualization (Fig. 1a).

In previous studies it was demonstrated that only the upper molecular weight band (47 kDa) of r*S.lividans*Apa was mannosylated [19]. To determine the relative proportion of each band, the purified doublet was analyzed using *ImageJ* software (https://imagej.nih.gov/ij/download.html). The results showed that both bands were found almost in the same relative concentration (Fig. 1b).

### Recombinant Apa Proteins from *S. lividans *and *E. coli* Induce a Decrease in CFU´s After Tuberculosis Aerosol Infection

**Fig. 2:**
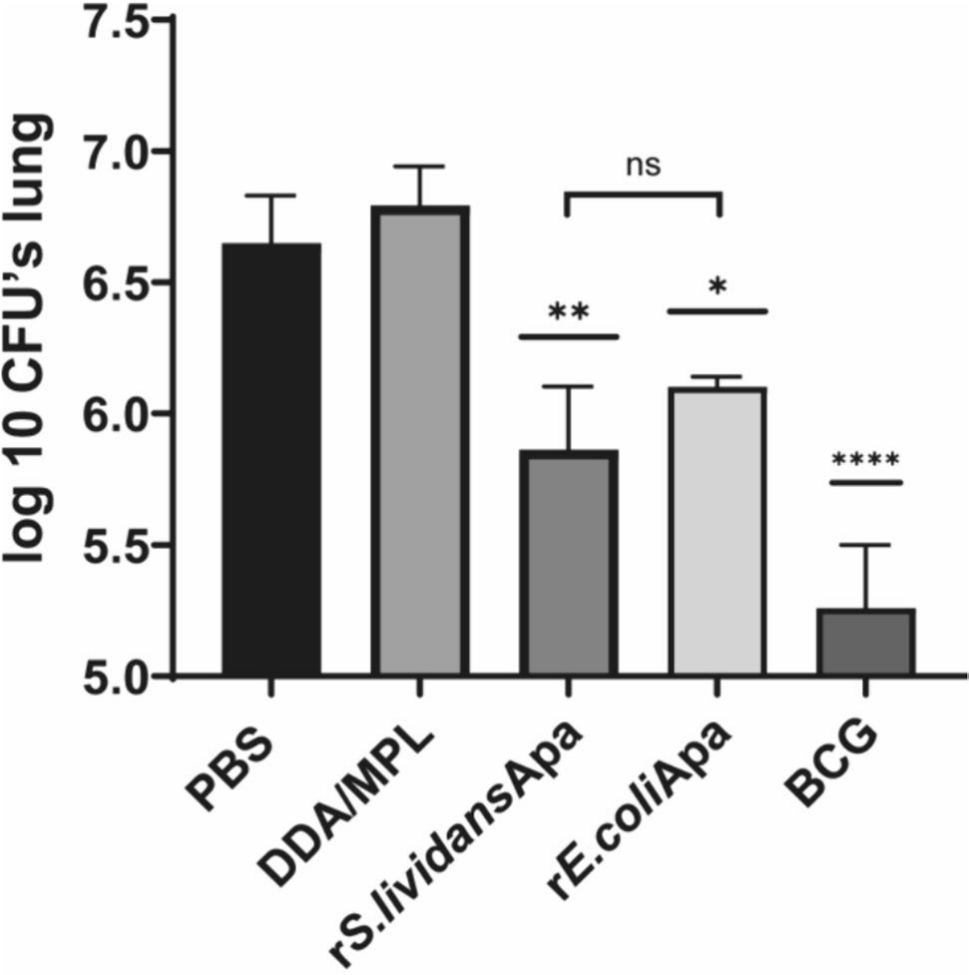
Protective response of intranasal administration of recombinant Apa proteins. Pulmonary bacterial load of *Mycobacterium tuberculosis* after intranasal administration of three doses of recombinant r*S.lividans*Apa and r*E.coli*Apa proteins in BALB/c mice compared to BCG vaccinated, adjuvant alone and non-inoculated controls. Four weeks after the last dose of recombinant Apa proteins or 8 weeks after one dose of BCG, all groups were challenged by aerosol route with virulent *M. tuberculosis* H37Rv. Six weeks post challenge, all mice were sacrificed and lung CFU´s quantified after 4 weeks of incubations at 37 °C. Numbers were expressed as the log_10_ values of the geometry mean for three mice. Data presented are representative of three similar experiments. Statistical comparisons among the groups were done by one way ANOVA and Tukey’s test. Significant differences are shown: *P < 0.05, **P < 0.01, ****P < 0.0001 with respect to controls

The capacity of the proteins to reduce the bacillary burden in mice infected with *M. tuberculosis* H37Rv via aerosol was evaluated after 6 weeks post-infection. As shown in Fig. 2, both proteins were able to induce a decrease the CFU’s in the infected animal lungs. Although the bacillary load reduction was lower than BCG, both peptide antigens forms, the glycosylated/non-glycosylated two bands protein mix had a protector effect regarding the unvaccinated control animals (Fig. 2).

### Lymphoproliferative T Cell Response to Recombinant *S. lividans*, *E. coli* Apa and BCG in Mice Inoculated with Antigens and Bacteria

**Fig. 3:**
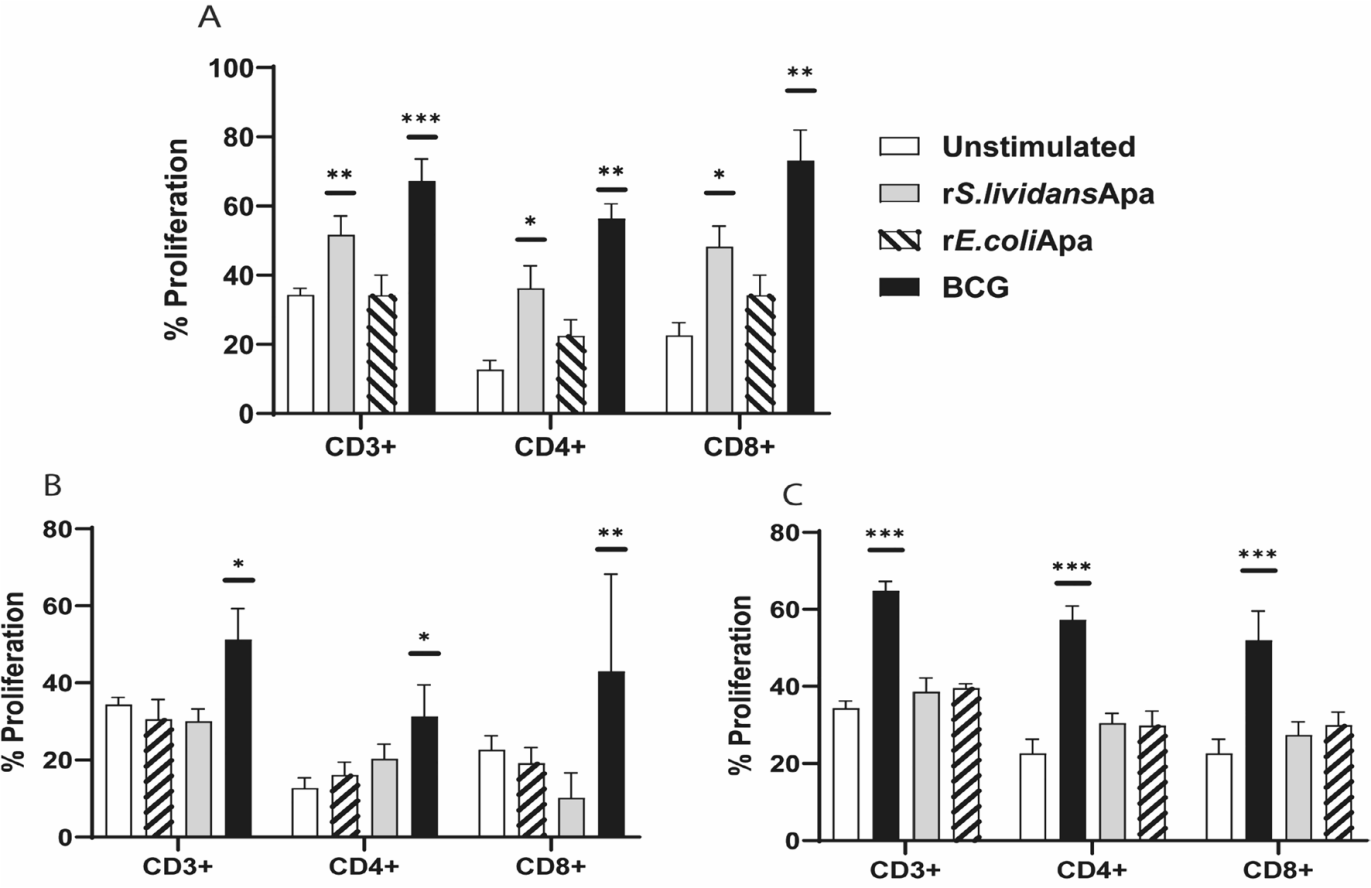
Recall of T cells (CD3+, CD4+ and CD8+) proliferative responses induced by r*S.lividans*Apa, r*E. coli*Apa and BCG. Proliferation of T cells populations were measured by flow cytometry*.* **a** Recall proliferative response of splenocytes from mice inoculated with r*S.lividans*Apa, (gray bar), cross-stimulation proliferative response of splenocytes stimulated with r*E.coli*Apa (diagonal stripes bar) and splenocytes stimulated with BCG (black bar). **b** Recall proliferative response of splenocytes from mice inoculated with r*E.coli*Apa (diagonal stripes bar), cross-stimulation response of r*S.lividans*Apa (gray bars) and splenocytes stimulated with BCG (black bars). **c** Recall proliferative response of splenocytes from mice inoculated with BCG (black bars), cross-stimulation response of splenocytes stimulated with r*S.lividans*Apa (gray bars) and splenocytes stimulated with r*E.coli*Apa (diagonal stripes bars). Negative control, proliferative response of splenocytes from mice inoculated with PBS (white bars). The results were calculated as means ± standard deviation of duplicated determinations of three different mice per group. The experiment is representative of two experiments. Statistical comparisons among the groups were done by one way ANOVA and Tukey´s test. Significant differences are shown: *P < 0.05, **P < 0.01 ***P < 0.001 with respect to control

To evaluate the immune response to recombinant proteins and BCG, cells from control and immunized mice were stimulated with PBS or with the corresponding recombinant Apa protein and BCG (Recall response). r*S.lividans*Apa and BCG induced a significative proliferation of CD3+, CD4+ and CD8+T cells (Fig. 3a, c), however, no proliferation of cells stimulated with r*E.coli*Apa was observed (Fig. 3b).

Cell proliferation response was also studied after cross-stimulation of cells from animals immunized with r*S.lividans*Apa, r*E.coli*Apa and BCG. Results showed that no proliferation of cells was observed in any case (Fig. 3b, c).

### Th1 Cytokine Response Induced by r*S.lividans*Apa, r*E.coli*Apa and BCG After Intranasal Administration of Proteins and Bacteria

Th1 pro-inflammatory cytokines were measured from supernatants of splenocytes from control groups and immunized mice stimulated with the corresponded recombinant Apa proteins or BCG.

**Fig. 4:**
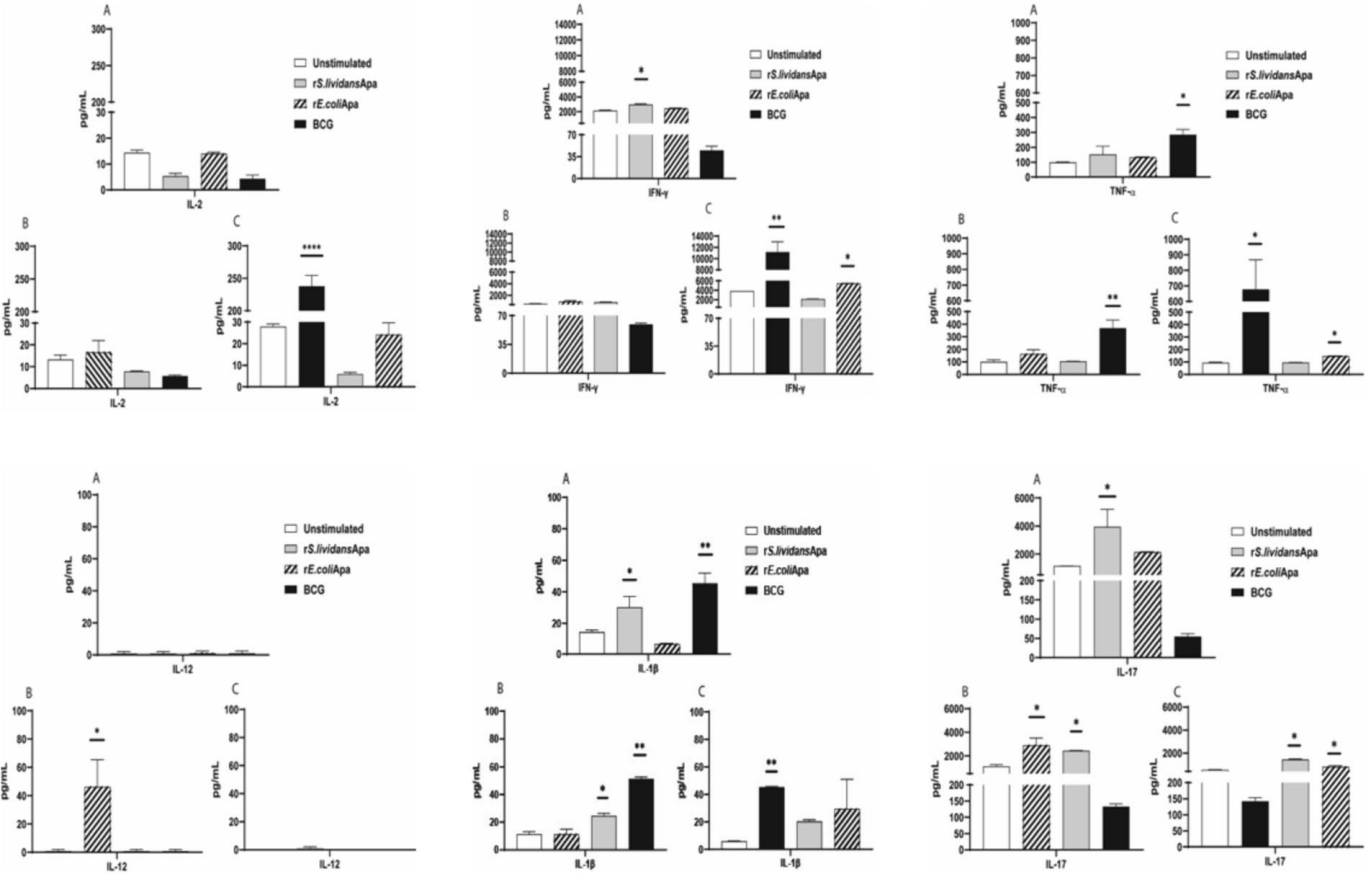
Th1 and Th17 cytokines induced by recombinant Apa proteins and BCG. Recall and cross stimulation responses induction of IL-2, INF-γ, TNF-α, IL-12 and IL-1β and IL-17. **a** Recall response of splenocytes from mice inoculated with r*S.lividans*Apa, (gray bar), cross-stimulation response of splenocytes stimulated with r*E.coli*Apa (diagonal stripes bar) and splenocytes stimulated with BCG (black bar). **b** Recall proliferative response of splenocytes from mice inoculated with r*E.coli*Apa (diagonal stripes bars), cross-stimulation response of splenocytes stimulated with r*S.lividans*Apa (gray bars) and splenocytes stimulated with BCG (black bars). **c** Recall response of splenocytes from mice inoculated with BCG (black bar), cross-stimulation response of splenocytes stimulated with r*S.lividans*Apa, (gray bar) and splenocytes stimulated with r*E.coli*Apa (diagonal stripes bar). Negative control, proliferative response of splenocytes from mice inoculated with PBS (white bars). The results are calculated as means ± standard deviation of duplicated determinations of pooled cells from three mice. Statistical comparisons among the groups were done by one way ANOVA and Tukey´s test. Significant differences are shown: *P < 0.05, **P < 0.01, ***P < 0.001 with respect to control

Specific T cell recall response was characterized by IFN-γ, IL-1β and IL-17 induction in response to r*S.lividans*Apa, Fig. 4a). In contrast IL-12 and IL-17 were produced in response to r*E.coli*Apa (Fig. 4b), and IL-2, IFN-γ, TNF-α and IL-1β in response to BCG stimulation (Fig. 4c).

Cytokine production was also studied after cross stimulation between of cells from animals immunized with r*S.lividans*Apa, r*E.coli*Apa and BCG. Results showed, that cross stimulation of cells from animals immunized with both recombinant proteins and stimulated BCG-induced TNF-α e IL-1β (Fig. 4a, b). On the other hand, cross-stimulation of cells from animals immunized with r*E.coli*Apa and stimulated with r*S.lividans*Apa induced IL-1β and IL-17 (Fig. 4b). In addition, cross-stimulation of cells from animals immunized with BCG and stimulated with *rE.coli*Apa induced a significant production of IFN-γ, TNF-α and IL-17. Also, cross-stimulation of cells from animals immunized with BCG and stimulated with r*S.lividans*Apa-induced IL-17 (Fig. 4c).

### IL-10 Cytokine Production Induced by r*S.lividans*Apa, r*E.coli*Apa and BCG Intranasal Administrated

**Fig. 5:**
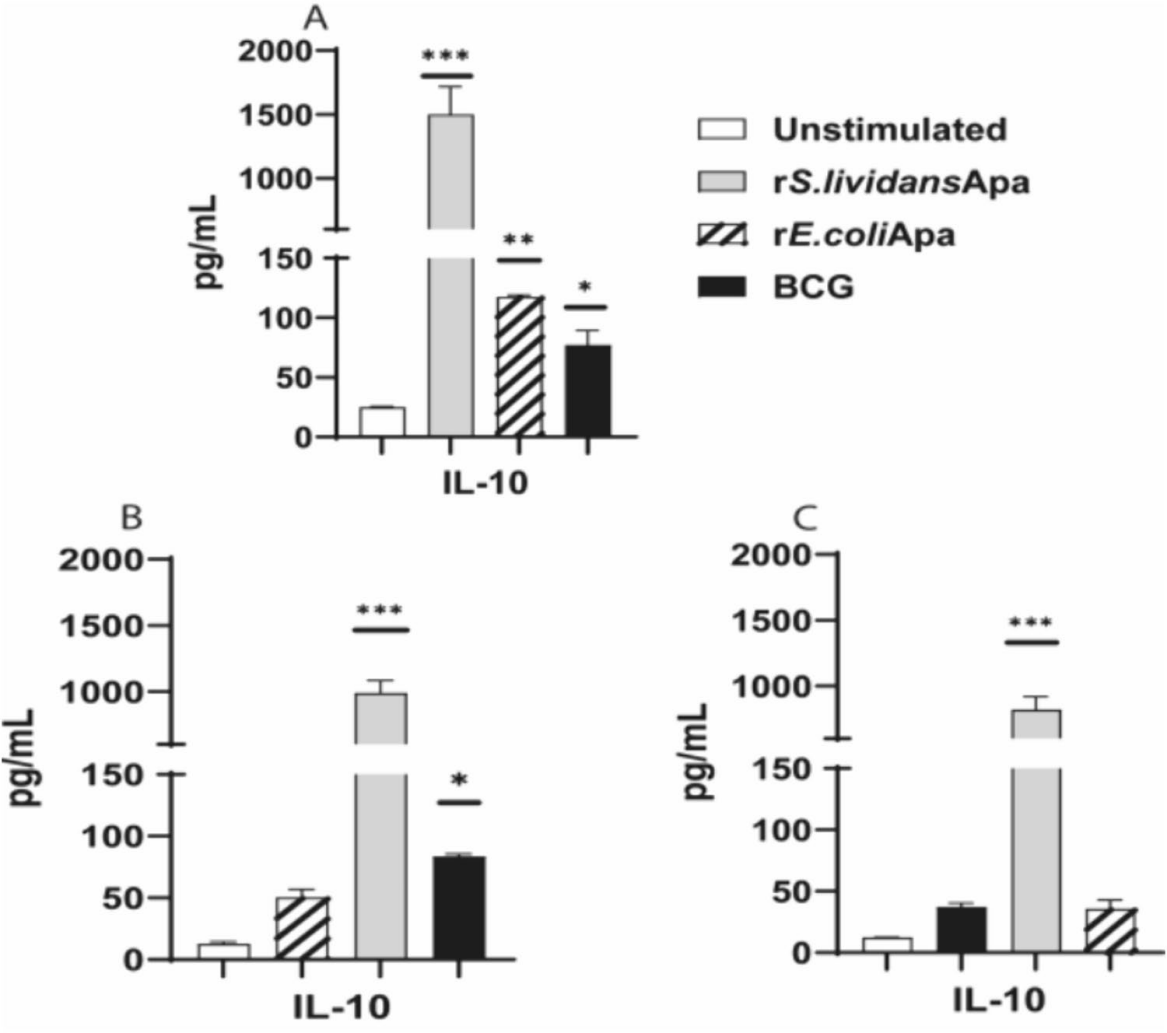
Th2 cytokine induced by recombinant Apa proteins and BCG. Recall and cross stimulation responses, induction of IL-10. **a** Recall cytokines response of splenocytes from mice inoculated with r*S.lividans*Apa (gray bar), cross-stimulation response of splenocytes stimulated with r*E.coli*Apa (diagonal stripes bar) and splenocytes stimulated with BCG (black bar). **b** Recall cytokines response of splenocytes from mice inoculated with r*E.coli*Apa (diagonal stripes bar), cross-stimulation response of splenocytes stimulated with r*S.lividans*Apa (gray bar) and splenocytes stimulated with BCG (black bar). **c** Recall response of splenocytes from mice inoculated with BCG (black bar), cross-stimulation response of splenocytes stimulated with r*S.lividans*Apa (gray bar) and splenocytes stimulated with r*E.coli*Apa (diagonal stripes bar). Negative control, cytokine response of splenocytes from mice inoculated with PBS (white bars). The results are calculated as means ± standard deviation of duplicated determinations of pooled cells from three mice. Statistical comparisons among the groups were done by one way ANOVA and Tukey´s test. Significant differences are shown: *P < 0.05, **P < 0.01, ***P < 0.001 with respect to control

The anti-inflammatory cytokine IL-10 was also evaluated. The Apa proteins an BCG recall response results for IL-10 are shown in Fig. 5, it is worth noting that only cells from animals immunized with r*S. lividans*Apa produced a high amount of IL-10 (Fig. 5a), in contrast with not induction of this cytokine by r*E.coli*Apa (Fig. 5b) and BCG (Fig. 5c). However, a significant production of this cytokine was observed after cross stimulation of cells from animals immunized with r*S.lividans*Apa and stimulated with r*E.coli*Apa and BCG (Fig. 5a). In the other hand, cross-stimulation of cells from animals immunized with r*E.coli*Apa and stimulated r*S.lividans*Apa-induced significant amounts of IL-10 (Fig. 5b). Finally cross stimulation of cells from animals immunized with BCG and stimulated whith r*S.lividans*Apa-induced IL-10 (Fig. 5c).

## Discussion

Tuberculosis studies on both infected human and experimental animals have shown that an effective immune response against *M. tuberculosis* involves the participation of macrophages, dendritic cells, alfa-beta (αβ), gamma-delta (γδ) cells, CD4+ and CD8+T lymphocytes and a broad diversity of cytokines produced by the immune cells [36, 37].

The protective immune response against *M. tuberculosis* is characterized by the activation of CD4+T helper type lymphocytes (Th1), that produce pro-inflammatory cytokines such as IFN-γ and TNF-α which can restrict the growth of tuberculous bacilli [38]. CD4+T lymphocytes Th17 type, which produce IL-17, are involved in the regulatory immune response [39]. Similarly, CD8+T lymphocytes produce IFN-γ and have an important role in inducing apoptosis of infected cells [40] and both CD4+ and CD8+T lymphocytes can produce pro and anti-inflammatory cytokines that play critical roles in macrophage activation and cell migration involved in formation of granulomas [41].

Together those observations make it clear that tuberculosis infection appears to be a major challenge because although the protective main mediators are induced during infection, they are not enough to combat the disease [42]. Furthermore, studies on tuberculosis vaccine development must consider the capacity of the candidate vaccine subunits to induce the different T type lymphocytes (Th1/Th2/Th17/Treg) in whose balance the generation of the protective immune response could reside. *M. tuberculosis* has a wide repertoire of antigenic proteins, some of them are being evaluated as subunit-booster vaccines after BCG vaccination, as these proteins could stimulate pre-existing-specific memory immune cells in response to BCG vaccination [42].

Among them, there are proteins modified by mannosylation, the presence of these molecules was evidenced in the, *M. tuberculosis* glycoproteome*,* where about 40 proteins from bacterial culture supernatant were identified by their ability to bind to lectin Con-A [43]. It is important to mention, that mannosylation patterns for most of the glycoproteins identified are unknow. In this sense, so far, the best studied glycoprotein is Apa, where mannosylation sites and mannose composition have been dilucidated [18]. Furthermore, it is important to mention that Apa is also one of the glycosylated proteins studied as subunit vaccine candidate. Studies have been performed to demonstrate the immunogenicity and antigenicity of native and non-glycosylated recombinant protein expressed in *E. coli* [12–16]. An interesting role for Apa glycosylation in *M. tuberculosis* virulence has also been suggested, as overexpression of native Apa in BCG has been shown to abrogate the protection conferred by BCG [44].

In this work, we studied the specific immune response generated against the r*S.lividans*Apa, a mixture of glycosylated/non-glycosylated protein, in splenocytes of BALB/c mice immunized i.n. with the protein and comparison with two controls, the BCG vaccine and the non-mannosylated r*E.coli*Apa protein, which has already been established as a protein capable of inducing protection [15, 16].

In addition, cross stimulation between the working groups was evaluated to look for synergy between the same proteins or between the proteins and BCG vaccine. Results showed that cells from animals vaccinated with either r*S.lividans*Apa or BCG, proliferated after stimulation with the homologous stimulus. On the contrary, no cell proliferation was observed when cells from animals vaccinated with r*E.coli*Apa were stimulated with this protein. This result is in agreement with the initial study by Romain et al. [14], who found that native non-glycosylated Apa was a 30-fold lower potency in inducing T lymphocyte proliferation and later Horn et al. [12], demonstrated the same phenomenon but with r*E.coli*Apa. Regarding cytokine production by r*S.lividans*Apa and r*E.coli*Apa as main vaccines, in this work we found that r*S.lividans*Apa, induced IFN-γ, IL-1β, IL-17 and IL-10 while IL-12 and IL-17 were induced by r*E.coli*Apa, and IFN-γ, IL-1β, IL-2 and TNF-α by BCG vaccine. It is worth noting that r*E.coli*Apa-induced IL-12 and IL-17 which are not classic markers of protection against tuberculosis [45].

On the other hand, the presence of IFN-γ in T-cell responses to protein-vaccine has been considered as an indicator of improved protection [10] and the induction of IL-1β and IL-17 by r*S.lividans*Apa could be also involved in protection against *M. tuberculosis* infection.

Furthermore, both r*E.coli*Apa and r*S.lividans*Apa-induced significant IL-17 production, suggesting this result that IL-17 could be produced independently of mannosylation. Taking these results together, one could hypothesize that proliferation of CD4+T lymphocytes together with IFN-γ and IL-17 are the necessary parameters for the reduction of CFU´s in the lung, considering the importance of these cytokines in the immune response to *M. tuberculosis* [46]. Cross stimulation of cells from animals immunized with r*S.lividans*Apa and stimulated with r*E.coli*Apa and vice versa had no impact on cell proliferation. However, a decrease in IFN-γ and IL-17 production was observed by cells from animals immunized with r*S.lividans*Apa and stimulated with r*E.coli*Apa. It was notable that IL-12 could not be detected, in contrast with the induction of IL-1β in cells from animals immunized with r*E.coli*Apa and stimulated with r*S.lividans*Apa suggesting this result that mannosylation could be inhibiting IL-12 and increasing production of IL-1β.

On the other hand, in cells from animals immunized with either, r*S.lividans*Apa or r*E.coli*Apa and stimulated with BCG, a decrease in IFN-γ and IL-17 was observed in both cases, together with the induction of TNF-α and IL-1β. In contrast, cells from animals immunized with BCG and stimulated with r*E.coli*Apa produced IFN-γ TNF-α and IL-17, while, reduction of IFN-γ and induction of IL-17 were observed after stimulation with r*S.lividans*Apa. Together those results show the importance of both the mannosylated and non-mannosylated Apa in the induction of the immune response.

With regard to cross stimulation in the case of Th2 response, it was observed that stimulation of cells from r*S.lividans*Apa immunized animals, with r*E.coli*Apa induced an environment with low IL-10 production, which increased drastically after of stimulation of cells from r*E.coli*Apa immunized animals with r*S.lividans*Apa showing the results that mannosylation induces IL-10, even in splenocytes stimulated with non-mannosylated protein. Furthermore, r*S*.*lividans*Apa-induced IL-10 in cells from animals immunized with BCG, in contrast to BCG recall response. Together these results, it is tempting to speculate, that mannosylation could be playing a role in the induction of anti-inflammatory cytokine IL-10 and that the increased production of this cytokine could be promoting an environment of homeostasis, regulating the inflammation induced by the pro-inflamatory cytokines [47]. These results show the importance of the presence of both mannosylated/non-mannosylated forms of r*S*.*lividans*Apa, in the induction of a protective immune response against *M. tuberculosis.*

Finally, it is important to mention that Apa interacts through its glycan structures with host receptors such as mannose receptor, the intercellular adhesion molecule 3 non-integrin (DC-SIGN) and the surfactant protein A. Those receptors bind to mycobacteria facilitating their entry into phagocytes and dendritic cells [15, 48, 49]. Taking those observations together, it is tempting to propose that Apa glycosylation could be driving the induction of a certain immune response through its interaction with these cellular receptors.

## Conclusions

The findings of this work revealed that both *M. tuberculosis* Apa expressed in *S. lividans* as a mixture of mannosylated/non-mannosylated and Apa, expressed in *E. coli* as non-mannosylated protein, were able to induce a decrease in lung CFU’s of *M. tuberculosis* infected mice. The results also showed that a balance between mannosylated versus non-mannosylated molecules could be and important parameter for the induction of a more effective protective immune response against *M. tuberculosis.* Furthermore, the cytokine immune response elicited by the r*S. lividans*Apa complex was different compared to the response induced by non-mannosylated protein. However, both the complex and non-mannosylated protein conferred protection against *M. tuberculosis* exposure, suggesting that different immunological mechanisms may be activated because of the presence or absence of glycosylation. However, more research in this field will be necessary to understand the role of mannosylation of *M. tuberculosis* glycoproteins in the context of host–pathogen interaction and immune response.

### Supplementary Information

Below is the link to the electronic supplementary material.

Supplementary file1 (DOCX 113 kb)
