# Supplementary material for: Immune Response to the Recombinant Apa Protein from Mycobacterium tuberculosis Expressed in Streptomyces lividans After Intranasal Administration in Mice. Induction of Protective Response to Tubercle Bacillus Aerosols Exposure
Source: Curr Microbiol. 2024 May 30;81(7):197. doi: 10.1007/s00284-024-03697-7 (PMC11139747; doi:10.1007/s00284-024-03697-7)
Supplement: Supplementary file 1 — Supplementary file1 (DOCX 113 kb) [file 284_2024_3697_MOESM1_ESM.docx]

**
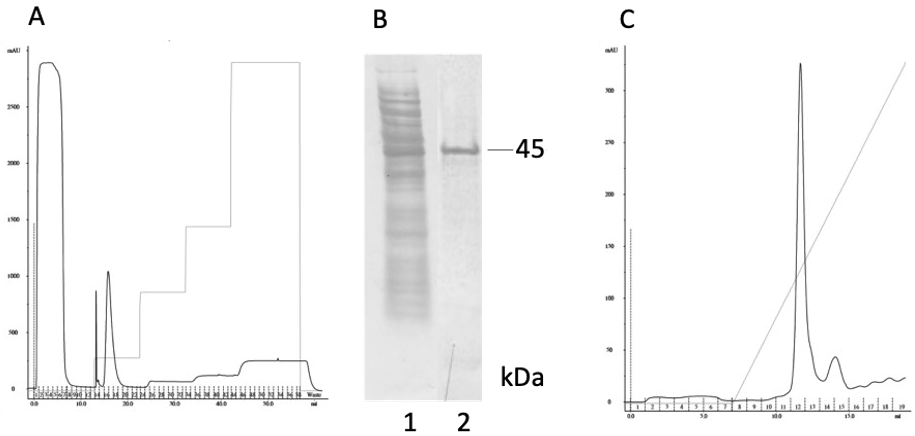
**

**Supplementary Figure.**

Purification of *rE.coliApa .*A) Chromatogram of protein purification from bacteria soluble extract by affinity HisTrap HP column B) Chromatogram of protein run in Sepharose HiTrap-Q anion exchange chromatography to eliminate LPS. C) SDS-PAGE of r*E.coliApa*. Lane 1, *E. coli* Soluble Extract and Lane 2, purified protein.
